# Identifying the silent deficiency: Severe Factor XI deficiency in an asymptomatic patient with isolated prolonged activated partial thromboplastin time—A case report

**DOI:** 10.1177/2050313X251401825

**Published:** 2025-12-22

**Authors:** Awni Alshurafa, Mahmoud Draidi, Feryal Ibrahim, Shehab F. Mohamed, Mohamed A. Yassin

**Affiliations:** 1 Hematology Department, Hamad Medical Corporation, Doha, Qatar; 2 Internal Medicine Department, Hamad Medical Corporation, Doha, Qatar; 3 Department of Laboratory Medicine and Pathology, National Center for Cancer Care and Research, Hamad Medical Corporation, Doha, Qatar

**Keywords:** Factor XI deficiency, prolonged PTT, coagulation disorder, inherited bleeding disorder, rare coagulopathy

## Abstract

Assessing a patient with a suspected bleeding disorder is a complex aspect of hematology. Prolonged activated partial thromboplastin time often indicates a coagulation factor deficiency or inhibitor and warrants detailed evaluation. Factor XI deficiency, a rare autosomal recessive disorder, shows variable bleeding tendencies with poor correlation between factor levels and bleeding risk. We report a 24-year-old asymptomatic male with an incidentally prolonged activated partial thromboplastin time during preoperative assessment for septoplasty. He had no bleeding or family history suggestive of a bleeding disorder. Mixing studies showed full correction, and factor assays confirmed severe Factor XI deficiency. Given the uncertain bleeding phenotype, a tailored perioperative plan was implemented, including fresh frozen plasma and prophylactic tranexamic acid. Recombinant activated Factor VII was available as rescue therapy, but not required. The surgery and postoperative course were uneventful, highlighting the importance of individualized perioperative management in Factor XI deficiency.

## Introduction

Evaluating a patient with a suspected bleeding disorder is a complex and challenging task in hematology. Initial assessment commonly includes coagulation tests such as prothrombin time (PT) and activated partial thromboplastin time (aPTT), which measure the time it takes for plasma to form a clot by generating thrombin. Prolongation of these times generally suggests a deficiency or the presence of an inhibitor. It is important to note that prolonged PT and aPTT do not always reflect an underlying bleeding tendency, highlighting the complexity of interpreting these results in clinical practice.^1,2^

A prolonged aPTT with a normal PT indicates a deficiency or inhibitor affecting the intrinsic pathway, such as Factors VIII, IX, or XI. Severe inherited Factor XI deficiency, characterized by Factor XI activity below 20%, is a rare condition with an estimated prevalence of approximately one in one million in the global population. As an autosomal disorder, it can affect both males and females.^3,4^ We report the case of a 24-year-old asymptomatic gentleman who was found to have a significantly prolonged aPTT during a preoperative evaluation. Further workup revealed severe Factor XI deficiency. Despite the severe deficiency and significantly prolonged aPTT, the patient was completely asymptomatic.

## Case presentation

A 24-year-old gentleman was referred to hematology following the incidental discovery of prolonged aPTT during preoperative assessment for deviated nasal septum correction. The patient’s medical history was significant for major depressive disorder, managed with sertraline monotherapy. He denied any other comorbidities, medication use, or substance exposure (including tobacco and alcohol). Of particular note was the consanguineous marriage of his parents (first cousins).

The patient reported no spontaneous bleeding episodes or thrombotic events throughout his life. Family history was negative for bleeding disorders, with no instances of easy bruising, prolonged bleeding after minor injuries, or excessive surgical bleeding in first-degree relatives. Physical examination revealed a morbidly obese but otherwise healthy-appearing male (weight 142 kg, height 175 cm, BMI 46 kg/m^2^). Vital signs were within normal limits. Comprehensive systemic examination demonstrated no abnormalities. There were no stigmata of bleeding disorders such as petechiae, ecchymosis, or hemarthroses.

Initial coagulation studies demonstrated an isolated prolongation of aPTT (Table 1). Subsequent evaluation included a mixing study, which showed complete correction of the prolonged aPTT both immediately and after 2-h incubation with normal pooled plasma, effectively ruling out an inhibitor. These findings were consistent with an intrinsic pathway factor deficiency. Factor-specific assays subsequently confirmed the diagnosis of Factor XI deficiency.

**Table 1.** Coagulation profile.

Blood test	Result (normal range)
PT	12.6 (9.4–12.5) s
INR	1
aPTT	98 (25.1–36.5) s
Factor VIII	131%
Factor IX	166.7%
Factor XI	<1% (70%–120%)
VWF AG	109.2%
VWF activity	113.9%

aPTT: activated partial thromboplastin time; PT: prothrombin time; INR: International Normalized Ratio; VWF AG: Von Willebrand Factor Antigen.

In anticipation of septoplasty surgery and considering the patient’s unknown bleeding phenotype—compounded by the lack of correlation between Factor XI levels and bleeding risk—a comprehensive perioperative hemostatic plan was implemented. The patient was admitted 48 h preoperatively for close monitoring and factor replacement. Fresh frozen plasma (FFP) was administered at 20 mL/kg ideal body weight, divided over 2 days to achieve adequate Factor XI levels while minimizing volume overload risk in this obese patient.

Prophylactic tranexamic acid (1 g every 8 h for 7 days) was initiated to provide additional hemostatic support through its antifibrinolytic activity. Recombinant activated Factor VII was made available as rescue therapy (30 µg/kg) for potential breakthrough bleeding, though its use was not required. Procedure bleeding was within the average.

The postoperative course was closely monitored with daily coagulation profiles and Factor XI level assessments (Table 2). This multidisciplinary approach ensured optimal hemostatic control throughout the perioperative period.

**Table 2.** aPTT and Factor XI result before and after surgery.

Labs	2 Days before^a^	1 Day before	Day of surgery	1 Day after	2 Days after
aPTT	94	61.1	49.6	53.5	55.4
Factor XI	<1	8	15.7	12.3	8.1

aPTT: activated partial thromboplastin time.

a Fresh frozen plasma was given after blood tests.

## Discussion

Factor XI deficiency, also referred to as Hemophilia C or Rosenthal syndrome, is a rare autosomal recessive bleeding disorder first described in 1953. It was initially described in patients who experienced excessive bleeding following dental extractions.^5^ Activation of Factor XI occurs via Factor XIIa on negatively charged surfaces through a process called contact activation. Factor XI plays a crucial role in thrombin generation and functions both as a procoagulant and an antifibrinolytic agent.^6^ Factor XI deficiency typically causes an isolated prolongation of the aPTT, although the correlation between Factor XI level and aPTT prolongation is weak; the aPTT usually becomes prolonged when Factor XI activity falls below ~40%–50%, and a normal aPTT does not exclude mild deficiency.

Unlike Hemophilia A and B, Hemophilia C is characterized by a distinct bleeding pattern, with most individuals not experiencing spontaneous bleeding, hemarthrosis, or muscle hematomas. Instead, bleeding in Factor XI deficiency is highly variable in both site and severity, typically occurring in response to trauma or surgery, particularly in tissues with high fibrinolytic activity. This unique pattern has led to the classification of Factor XI deficiency as an “injury-related bleeding disorder.”^7^

The reason behind the absence of spontaneous bleeding in individuals with low Factor XI levels lies in the minimal role of Factor XI in initiating the coagulation cascade. Clot formation begins with an initial burst of thrombin generated by the interaction of circulating Factor VIIa and tissue factor, exposed on injured endothelium. Factor XI is activated by this thrombin burst and amplifies clot formation by converting Factors IX to IXa, which activates Factor X and enhances thrombin generation through a positive feedback loop. This amplification stabilizes and maintains the clot, highlighting Factor XI’s role in clot reinforcement rather than initiation. As a result, spontaneous bleeding is uncommon, but a deficiency may lead to bleeding in situations requiring robust clot maintenance, such as surgery or trauma.^8,9^

Although bleeding is more frequent in individuals with Factor XI levels below 20%, the correlation between Factor XI activity and bleeding risk remains weak. A study by the European Network of Rare Bleeding Disorders found that, among all rare coagulation factor deficiencies, Factor XI exhibited the weakest association between factor levels and clinical bleeding manifestations.^10^ Supporting this, an analysis of 169 individuals from 24 families highlighted considerable variability in bleeding tendencies, even among those likely carrying the same Factor XI mutation. Remarkably, one-third of individuals with Factor XI levels above 20% experienced excessive bleeding during a surgical procedure.^11^

Preoperative prophylactic replacement of Factor XI is generally required for individuals undergoing major surgery if they have a significant bleeding history or if their bleeding history is unknown and their Factor XI level is below 20%. Replacement options include Factor XI concentrate or FFP, chosen based on availability and patient-specific considerations. Additional preoperative preparations may include antifibrinolytic therapy, such as tranexamic acid, to reduce perioperative bleeding, optimization of other coagulation factors if deficiencies are identified, and ensuring the availability of blood products for transfusion if necessary.^12–14^ In our patient, Factor XI concentrate was unavailable; therefore, FFP was used. Due to the patient’s morbid obesity and the long half-life of Factor XI, the plasma was administered in divided doses over 2 days to ensure optimal coverage and minimize risks.

This case highlights the importance of recognizing the variability in bleeding tendencies among individuals with severe Factor XI deficiency, which may present asymptomatically despite markedly prolonged aPTT. Preoperative planning with appropriate replacement therapy and antifibrinolytics is crucial in ensuring safe outcomes.
